# The Wisconsin Hydrocephalus Survey: shunt-dependent hydrocephalus management style among members of the American Society of Pediatric Neurosurgeons

**DOI:** 10.1186/2045-8118-12-S1-O12

**Published:** 2015-09-18

**Authors:** Mark Richard Kraemer, Bermans J Iskandar

**Affiliations:** 1 Department of Neurological Surgery, University of Wisconsin School of Medicine and Public Health, USA

## Introduction

This survey sought to evaluate differences in the understanding and management of shunt-dependent hydrocephalus among the senior North American Pediatric Neurosurgery membership.

## Methods

Surveys were sent to all active American Society of Pediatric Neurosurgeons (ASPN) members from September to November 2014. A total of 204 surveys were sent from which 130 responses were recorded, representing 64% of active ASPN membership. Respondents were asked 13 multiple choice and free response questions focusing on four problems encountered in shunted hydrocephalus management: Shunt malfunction, cerebrospinal fluid (CSF) overdrainage, chronic headaches and slit ventricle syndrome (SVS). Qualtrics® online survey software was used to distribute and collect response data.

## Results

ASPN surgeons prefer three varieties of shunt valves: 41% differential pressure, 29% differential with anti-siphon device (ASD), and 27% programmable. Respondents agree shunt obstruction occurs most often at the ventricular catheter due to either in-growth of the choroid plexus (67%), CSF debris (18%), ventricular collapse (8%), or other reasons (9%). Underlying causes of obstruction were attributed to small ventricular size, catheter position, choroid plexus migration, build-up of cellular debris, inflammatory processes, or CSF overdrainage. The majority of respondents (>85%) consider chronic overdrainage a rare complication. These cases are most often managed with ASDs or programmable valves. Chronic headaches are most often attributed to medical reasons (e.g. migraines, tension) and managed with patient reassurance. The most popular treatments for SVS include shunt revision (88%), cranial expansion (57%) and placement of an ASD (53%). SVS etiology was most often linked to early onset of shunting, chronic overdrainage and/or loss of brain compliance.

## Conclusions

This survey shows discrepancies in shunt-dependent hydrocephalus understanding and management style among a representative group of experienced North American pediatric neurosurgeons. In particular, there are differing opinions regarding the primary cause of ventricular shunt obstructions and the origins of SVS. However, there appears to be general consensus in approach and management of overdrainage and chronic headaches. These results provide impetus for better studies evaluating the pathophysiology and prevention of shunt obstruction and SVS.
